# Supplementary figures and images for: Gut commensal Enterocloster species host inoviruses that are secreted in vitro and in vivo
Source: Microbiome. 2023 Mar 30;11:65. doi: 10.1186/s40168-023-01496-z (PMC10061712; doi:10.1186/s40168-023-01496-z)

Bacteroidetes

49

Actinobacteria

16

Verrucomicrobia (1)

11

Proteobacteria

Fusobacteria (3)

Firmicutes

83

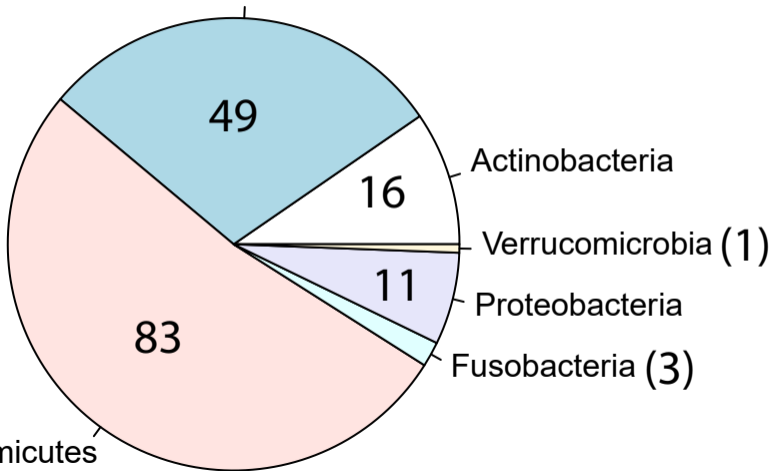

Supplement: Supplementary file 2 — Additional file 1: Figure S1. Pie chart of the phyla represented by the 163 gut bacteria screened with Inovirus detector. See Table S1 for the complete list of bacteria. [file 40168_2023_1496_MOESM1_ESM.pdf]

Average Nucleotide Identity

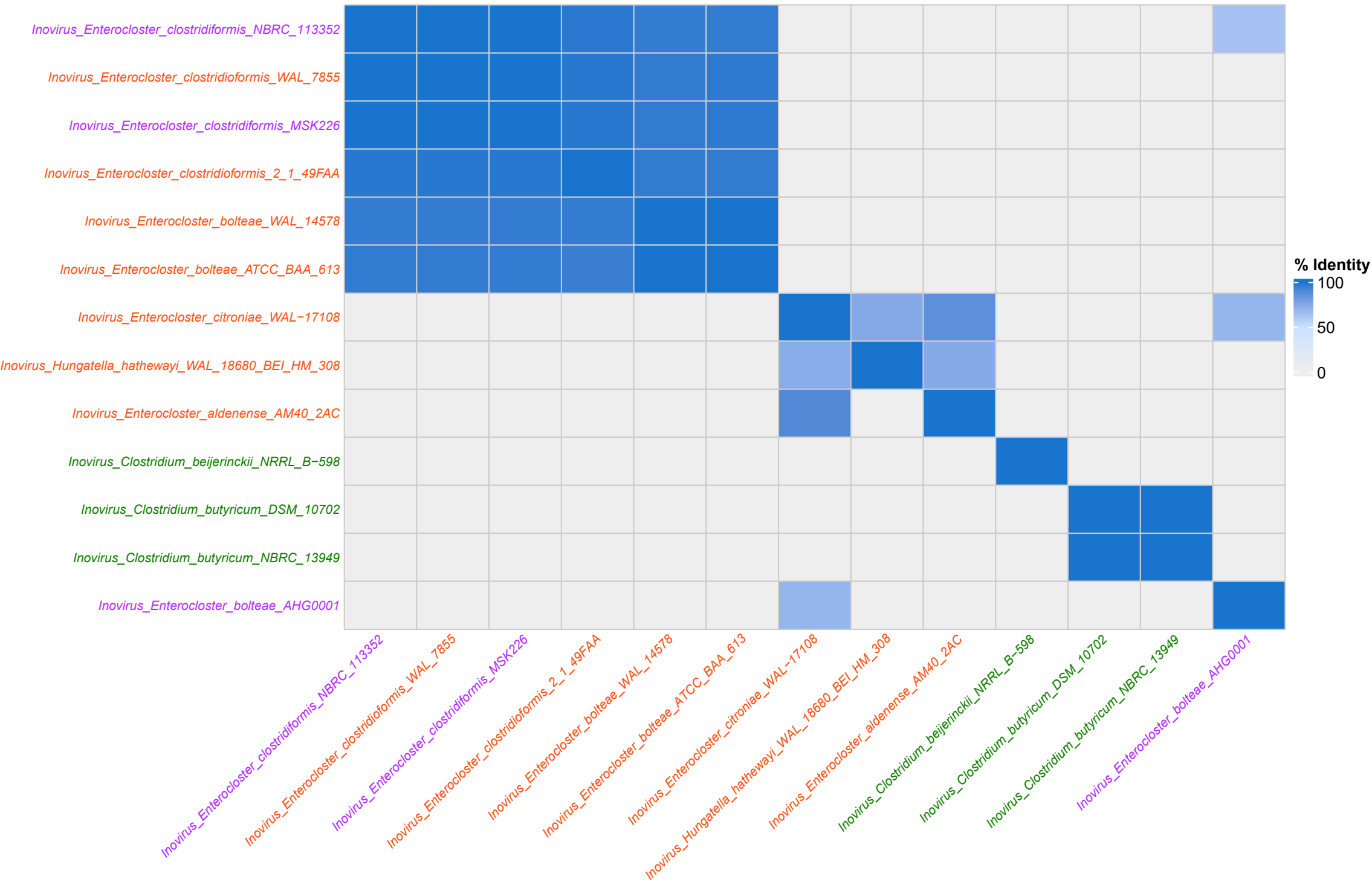

Supplement: Supplementary file 3 — Additional file 2: Figure S2. Expanded ANI analysis of inoviruses. Average nucleotide Identity (ANI) comparison of putative inovirus genomes (Table S2). This comparison includes identified inovirus genomes found after screening the Clostridium (green) and Enterocloster (purple) genomes in Table S3, as well are the original inoviruses we identified (red) (Fig. 1B). To further screen the Clostridium and Enterocloster inovirus genomes against a larger reference database, we performed nucleotide BLAST searches of the inovirus genomes and tBLASTx searches of their ORFs (results not shown). Other than to themselves, the BLAST matches were not significant, indicating a high specificity of these inoviruses for their strains. [file 40168_2023_1496_MOESM2_ESM.pdf]

Average Nucleotide Identity

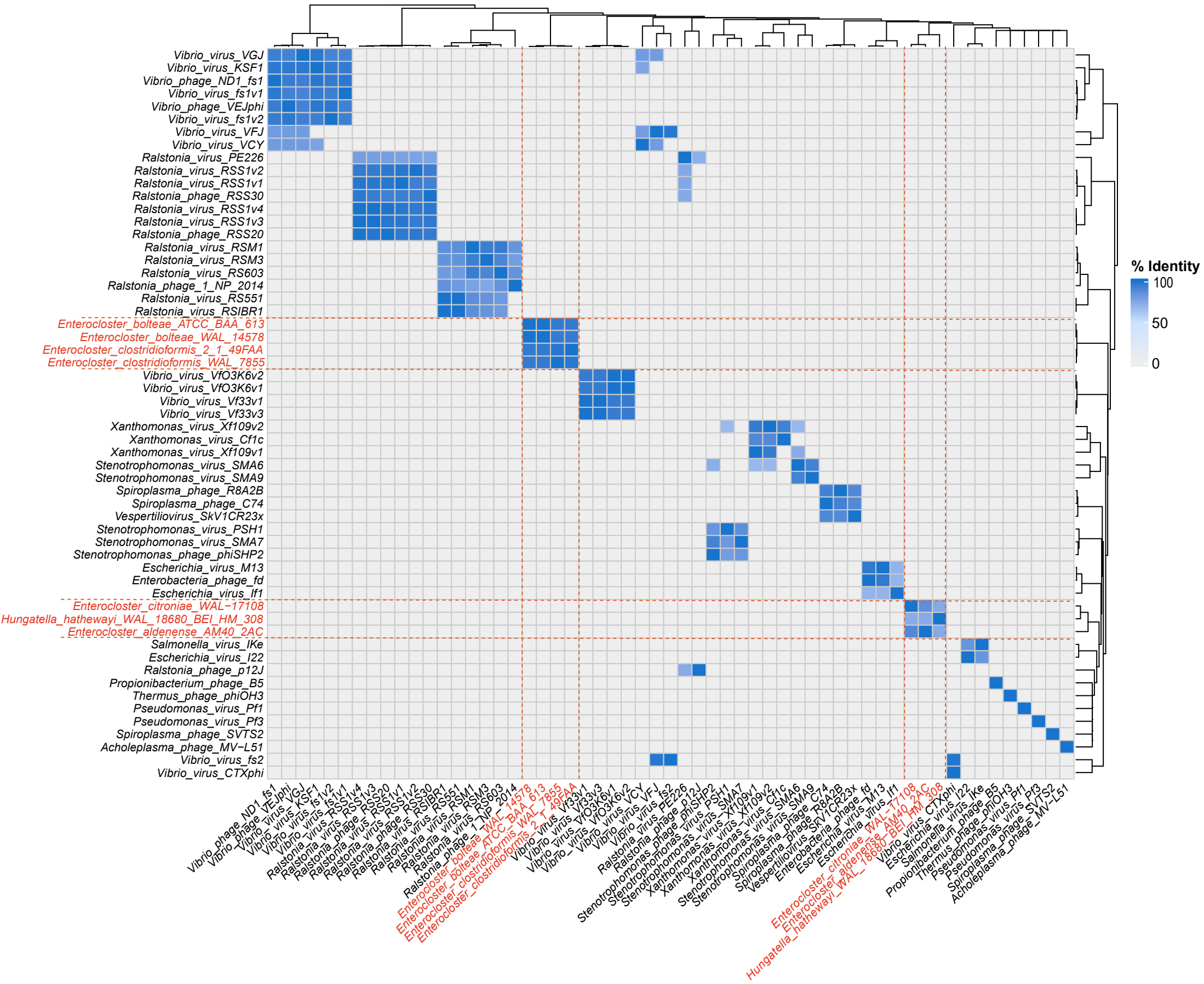

Supplement: Supplementary file 4 — Additional file 3: Figure S3. Putative inoviruses share no sequence identity to other reference inoviruses. ANI comparison of 45 inovirus genomes (Table S3) downloaded from NCBI and predicted inoviruses found in this study (highlighted in red). [file 40168_2023_1496_MOESM3_ESM.pdf]

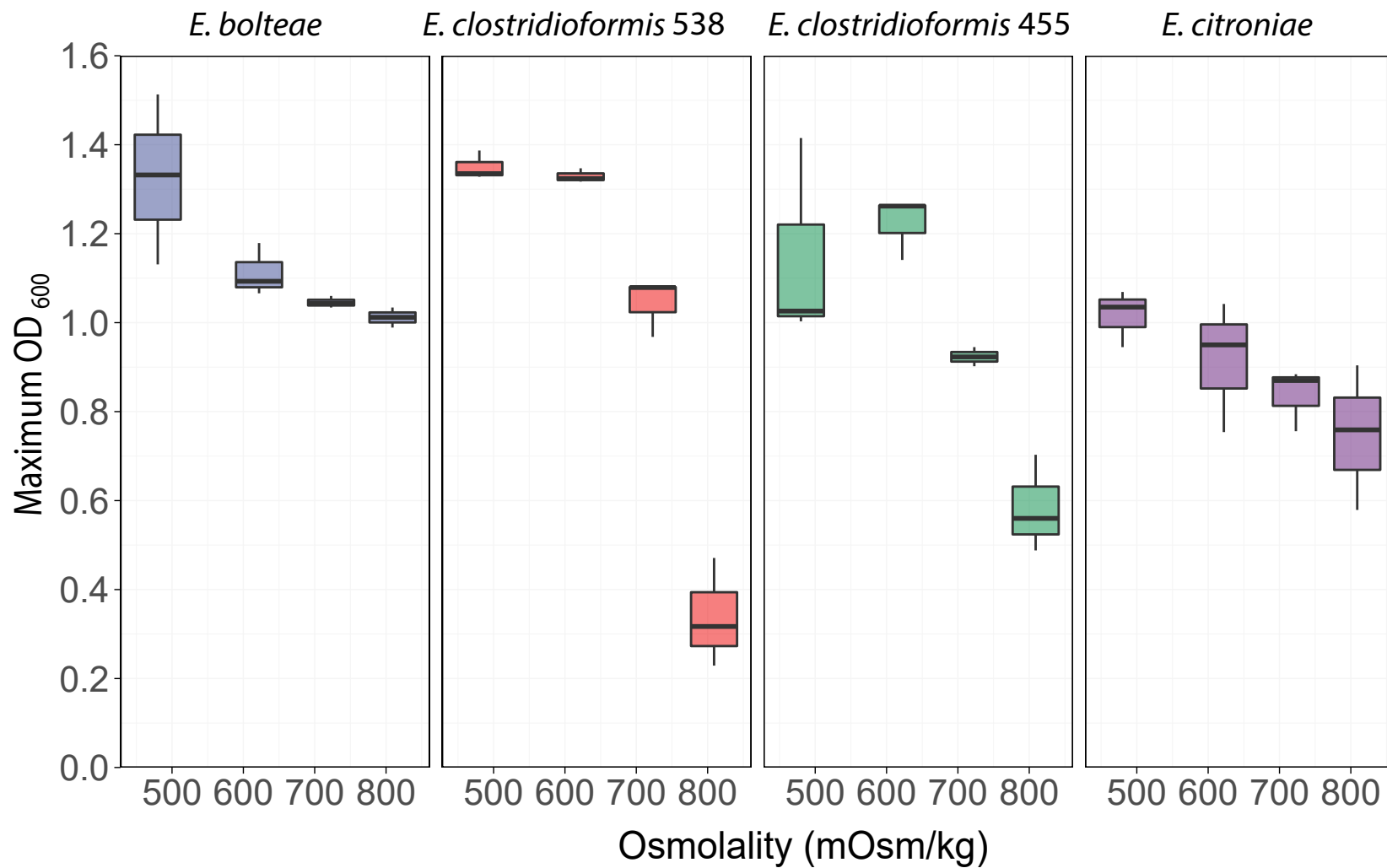

Supplement: Supplementary file 6 — Additional file 5: Figure S5. Overall maximum OD600 of Enterocloster spp. change in a strain-specific manner in response to osmotic stress. Maximum OD600 of selected Enterocloster strains was calculated from the growth curves obtained as explained in Figure 3A-B. Under different osmotic stresses, we observed varying bacterial sensitivity to osmolality at both the strain and species levels. Notably, we saw a decrease in growth rate (Fig. 3E) and overall max OD when osmolality was increased above the baseline media osmolality of 480 mOsm/kg in E. clostridioformis 538 and E. citroniae. Unlike its counterpart strain, we noticed that the slower-growing E. clostridioformis 455 exhibited tolerance to increased osmolality up to 722 mOsm/kg in the form of stable growth rates, despite decreasing overall max OD (Fig. 3E). Uniquely, E. bolteae exhibited faster growth rates only at 622 mOsm/kg (Fig. 3E), suggesting an optimal osmolality, despite declining overall max OD (Fig. S3). Nonetheless, E. bolteae also showed the overall highest tolerance to osmolality from all tested strains, as both its growth rate and max OD changed the least compared to baseline. These results highlight the unique relationships that bacteria, even at the species or substrain level, have with osmolality, which is consistent with previous findings in other gut bacteria [32]. [file 40168_2023_1496_MOESM5_ESM.pdf]

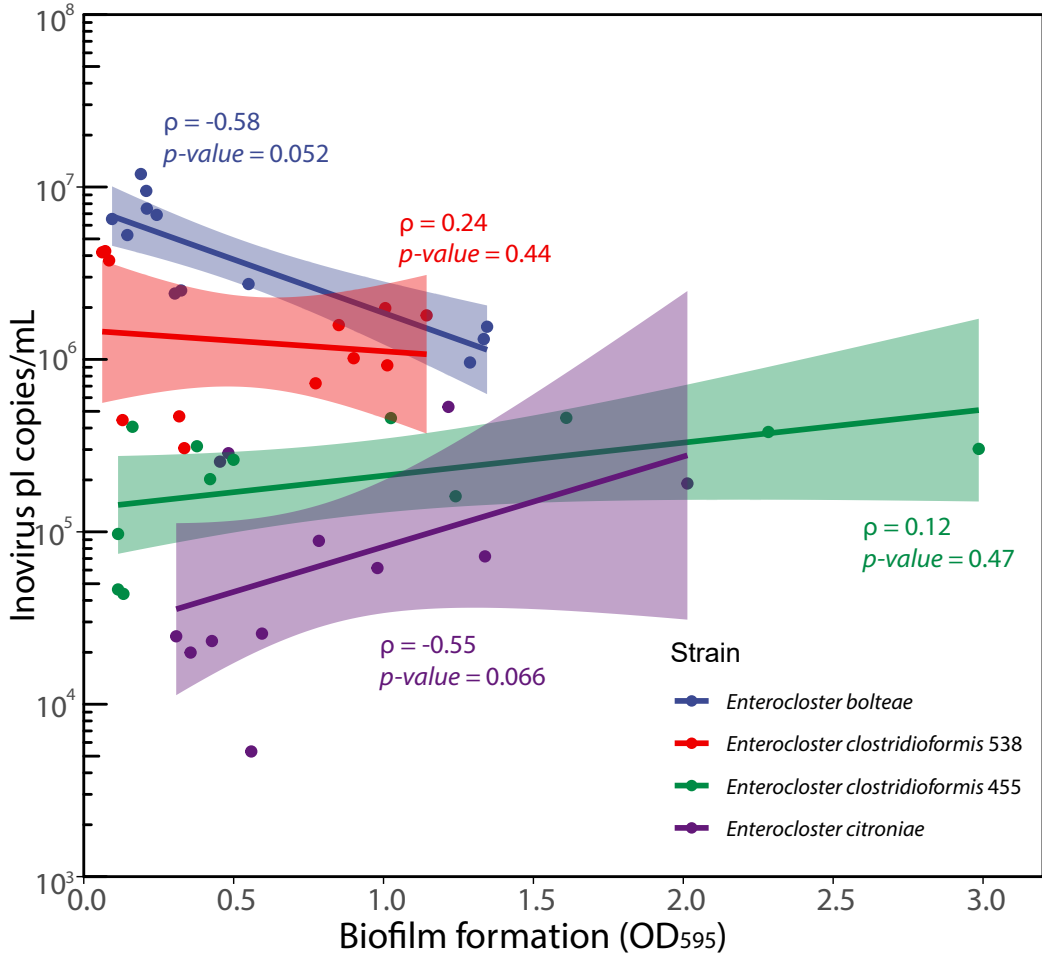

Supplement: Supplementary file 7 — Additional file 6: Figure S6. Inovirus secretion is not correlated with biofilm formation in Enterocloster strains. Linear regression and 95% confidence intervals are shown for each strain. Correlation coefficients and p-values obtained using Spearman's correlation. [file 40168_2023_1496_MOESM6_ESM.pdf]

**A**

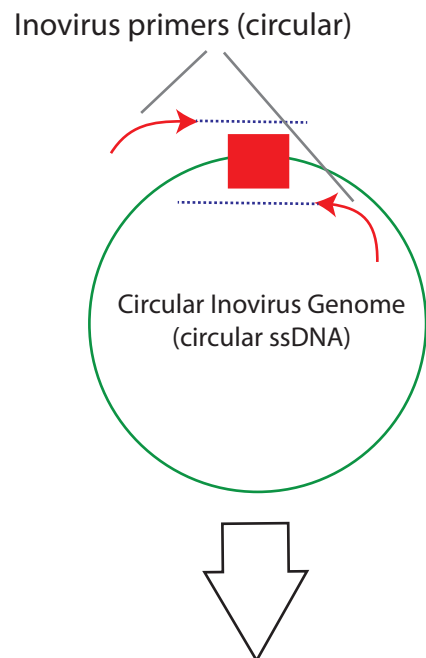

**B**

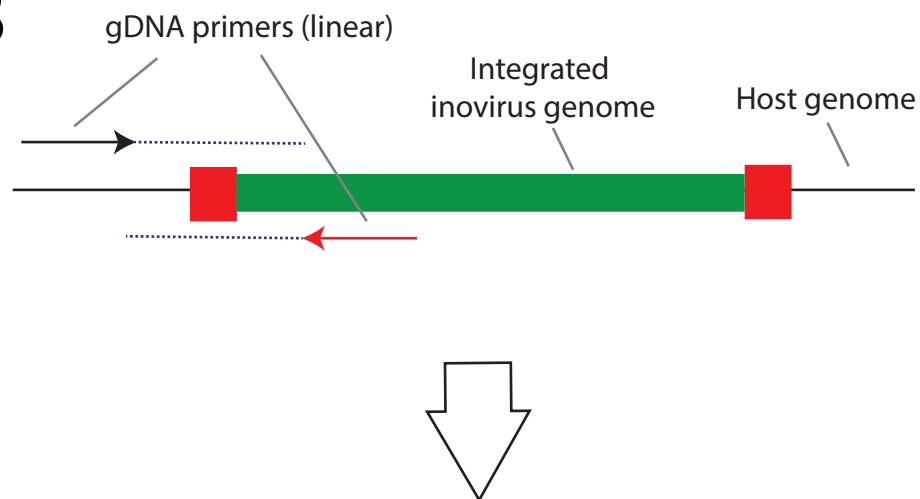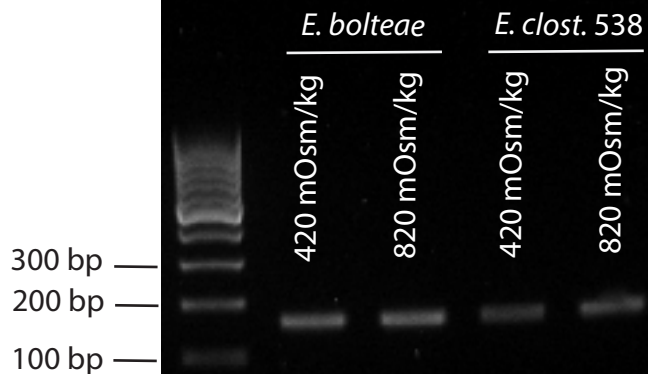

300 bp —  
200 bp —  
100 bp —

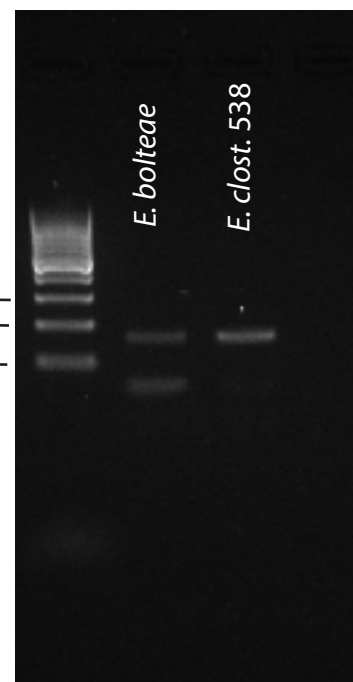

Supplement: Supplementary file 9 — Additiobal file 8: Figure S8. Validation of in vivo qPCR primers. A Top: diagram depicting the circularization region targeted by primers to detect inovirus genomes. Bottom: total DNA was extracted from stable phase Enterocloster cultures grown at the highlighted media osmolality. Extracted DNA was used as a template in PCR reactions containing the inovirus-specific primers. The resulting PCR product was resolved in a SYBR-stained 1% agarose gel. A 100 bp ladder was run in parallel to determine product size. B Top: diagram depicting the integration region amplified by primers that target the inovirus integrated genome and the host genome to quantify gDNA copies. Bottom: total DNA was extracted from stable phase Enterocloster cultures grown at base osmolality. Extracted DNA was used as a template in PCR reactions containing the gDNA primers. The resulting PCR product was resolved in a SYBR-stained 1% agarose gel. A 100 bp ladder was run in parallel to determine product size. [file 40168_2023_1496_MOESM8_ESM.pdf]

A

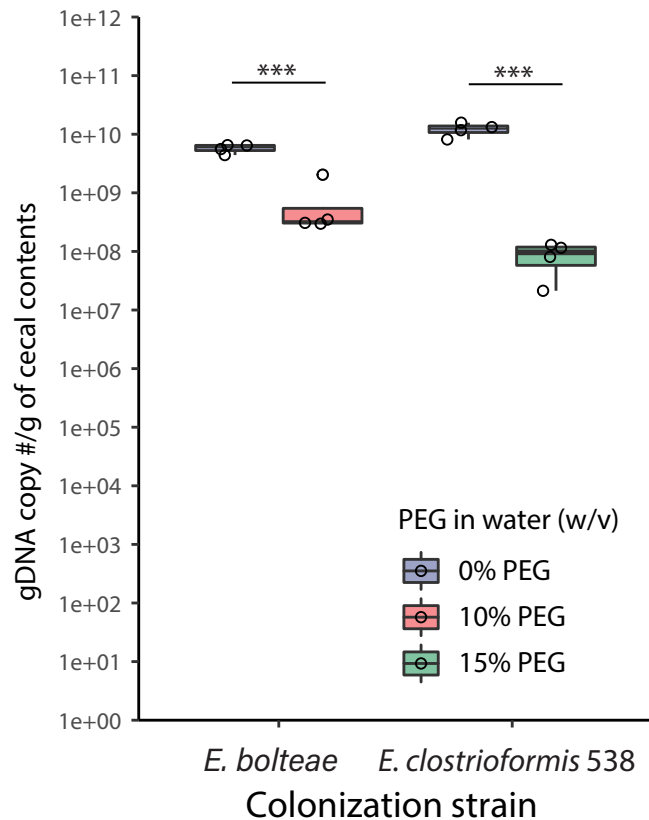

B

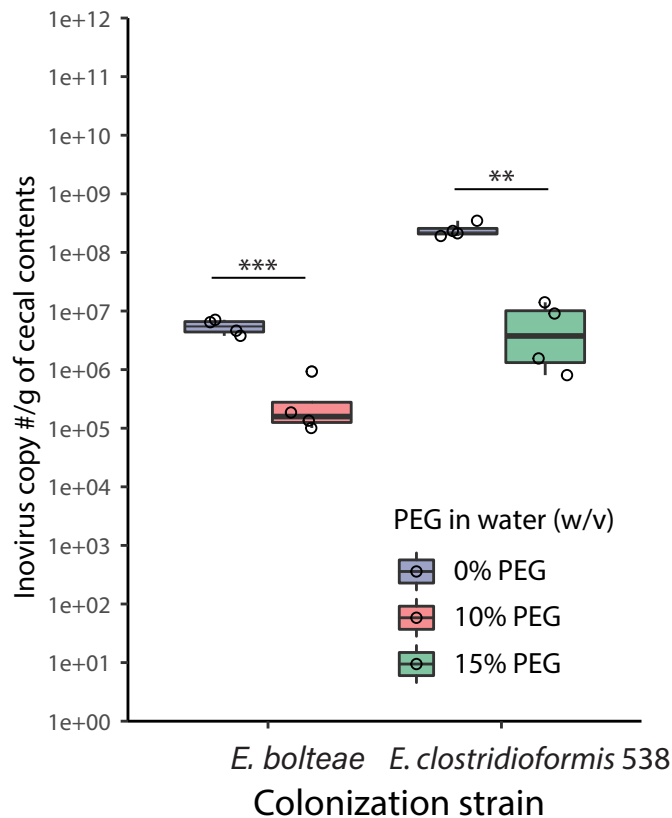

Supplement: Supplementary file 10 — Additional file 9: Figure S9. Bacterial and inovirus genome copies decrease in vivo after PEG treatment. A Bacterial gDNA copies and B Inovirus genomes copies quantified from cecal contents using absolute qPCR. To test for significance, a Student’s t-test was performed between each experimental group and their control after accounting for group variances using Bartlett's test; ** p < 0.01, ** p < 0.001. [file 40168_2023_1496_MOESM9_ESM.pdf]
