# Supplementary material for: Gut commensal Enterocloster species host inoviruses that are secreted in vitro and in vivo
Source: Microbiome. 2023 Mar 30;11:65. doi: 10.1186/s40168-023-01496-z (PMC10061712; doi:10.1186/s40168-023-01496-z)
Supplement: Supplementary file 5 — Additional file 4: Figure S4. Inoviruses are secreted in vitro. A Schematic demonstrating how inoviruses are detected in vitro. First, three-day-old cultures were separated to yield cell-free spent media supernatants that were filter-sterilized and then treated with DNase to eliminate contaminating bacterial DNA. The untreated (and unfiltered) and DNase-treated supernatants were then used as template DNA in qPCR and PCR reactions using primers targeting the pI gene and 16S rRNA gene to guarantee that there was no 16S contamination. qPCR assay using B pI and C 16S rRNA gene-specific primers in untreated and treated supernatants of Enterocloster cultures. Data shown for three biological replicates. Gel electrophoresis of PCR products from untreated and treated supernatants and D pI and E) 16S rRNA gene-specific primers. [file 40168_2023_1496_MOESM4_ESM.pdf]

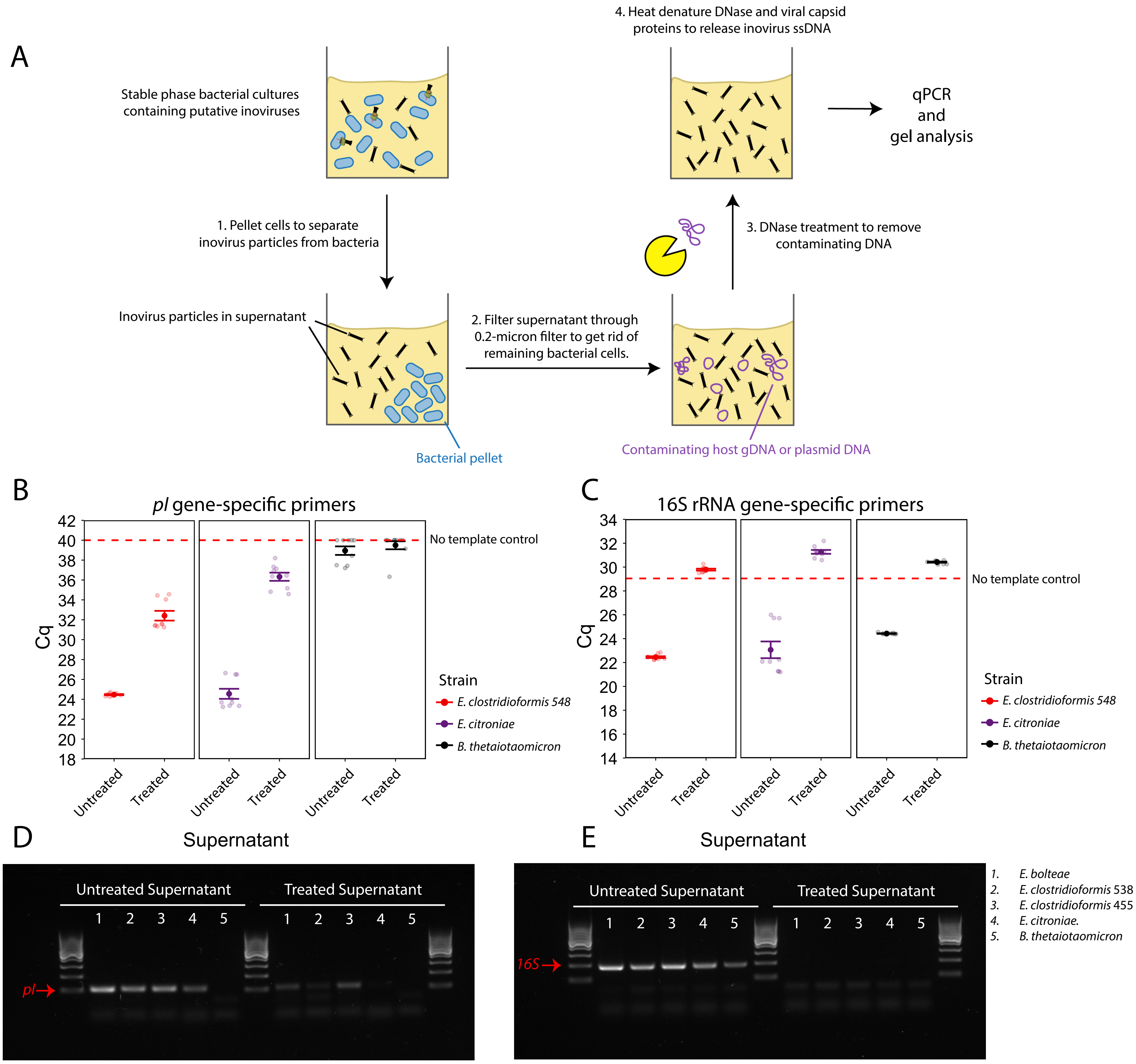

A
Stable phase bacterial cultures containing putative inoviruses
1. Pellet cells to separate inovirus particles from bacteria
Inovirus particles in supernatant
Bacterial pellet
2. Filter supernatant through 0.2-micron filter to get rid of remaining bacterial cells.
Contaminating host gDNA or plasmid DNA
3. DNase treatment to remove contaminating DNA
4. Heat denature DNase and viral capsid proteins to release inovirus ssDNA
qPCR and gel analysis
B
pI gene-specific primers
Cq
No template control
Untreated
Treated
Strain
E. clostridioformis 548
E. citroniae
B. thetaiotaomicron
C
16S rRNA gene-specific primers
D
Supernatant
Untreated Supernatant
Treated Supernatant
1 2 3 4 5
pI
E
16S
1. E. bolteae
2. E. clostridioformis 538
3. E. clostridioformis 455
4. E. citroniae.
5. B. thetaiotaomicron
