# Supplementary material for: Gut commensal Enterocloster species host inoviruses that are secreted in vitro and in vivo
Source: Microbiome. 2023 Mar 30;11:65. doi: 10.1186/s40168-023-01496-z (PMC10061712; doi:10.1186/s40168-023-01496-z)
Supplement: Supplementary file 8 — Additional file 7: Figure S7. E. bolteae and E. clostridioformis 538 share significant amino acid contents of ORFs. Top: protein blast (blastp) comparisons of E. bolteae translated ORFs against E. clostridioformis 538 translated ORFs; blastp top hit comparisons are depicted in the diagram with double-headed arrows. Bottom: resulting percentage identity and alignment coverage of blastp results. Row labels were assigned based on the annotations predicted in Figure 1C. [file 40168_2023_1496_MOESM7_ESM.pdf]

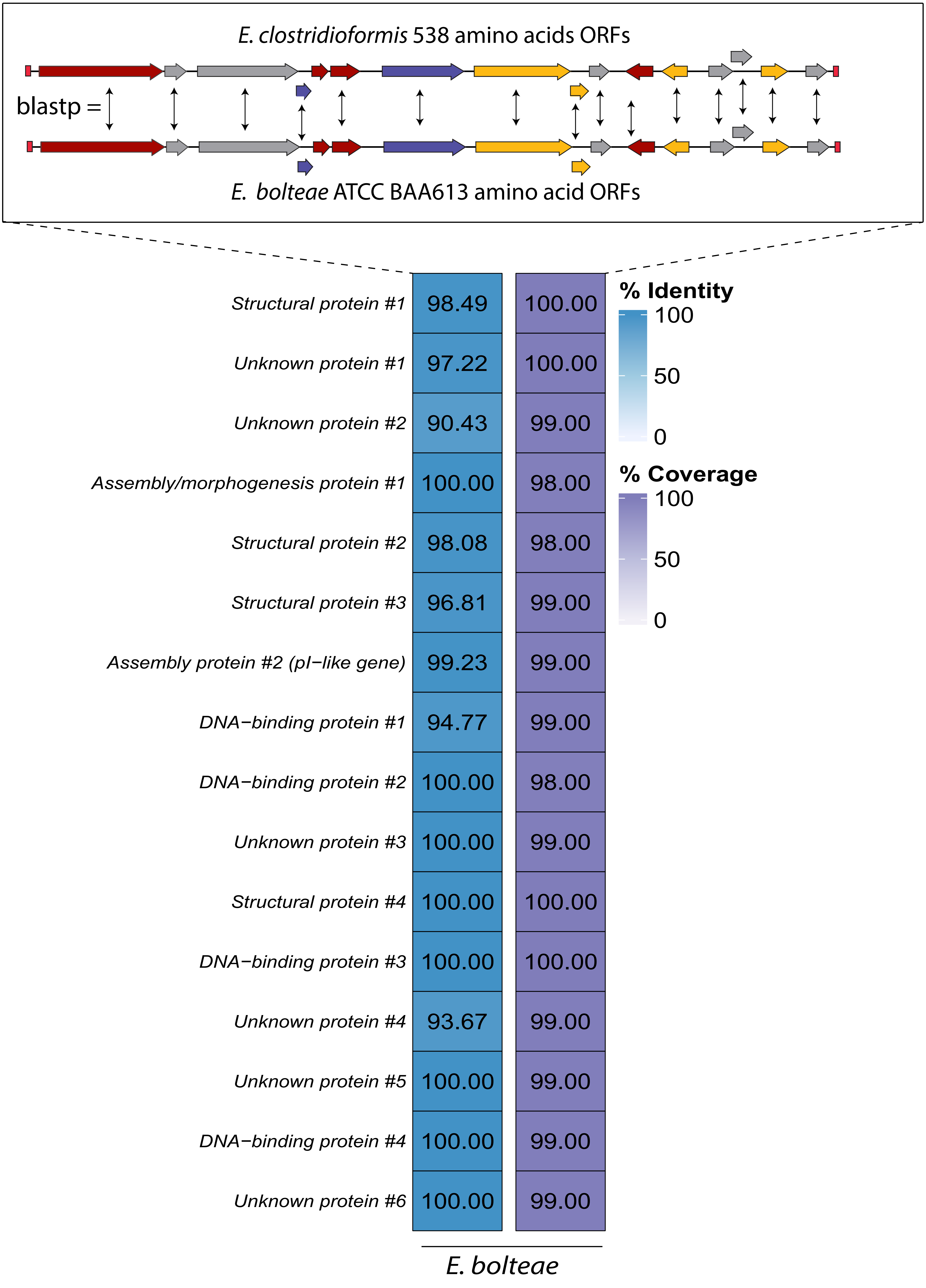
E. clostridioformis 538 amino acids ORFs
blastp =
E. bolteae ATCC BAA613 amino acid ORFs
Structural protein #1
98.49
100.00
Unknown protein #1
97.22
100.00
Unknown protein #2
90.43
99.00
Assembly/morphogenesis protein #1
100.00
98.00
Structural protein #2
98.08
98.00
Structural protein #3
96.81
99.00
Assembly protein #2 (pI−like gene)
99.23
99.00
DNA−binding protein #1
94.77
99.00
DNA−binding protein #2
100.00
98.00
Unknown protein #3
100.00
99.00
Structural protein #4
100.00
100.00
DNA−binding protein #3
100.00
100.00
Unknown protein #4
93.67
99.00
Unknown protein #5
100.00
99.00
DNA−binding protein #4
100.00
99.00
Unknown protein #6
100.00
99.00
% Identity
100
50
0
% Coverage
100
50
0
E. bolteae
